# Psychometric properties of the German version of the MacNew heart disease health-related quality of life questionnaire

**DOI:** 10.1186/1477-7525-10-83

**Published:** 2012-07-20

**Authors:** Lukas Gramm, Erik Farin, Wilfried H Jaeckel

**Affiliations:** 1 Department of Quality Management and Social Medicine (AQMS), University Medical Center Freiburg, Engelbergerstrasse 21, 79106, Freiburg, Germany; 2 Hochrhein-Institute for Rehabilitation Research, Bergseestrasse 61, 79713, Bad Saeckingen, Germany

## Abstract

**Background:**

There is a lack of German-language, disease-specific health related quality of life instruments applicable in cardiac rehabilitation. The purpose of this project was to investigate the psychometric properties of the German version of the MacNew Heart Disease Health-related Quality of Life Questionnaire (MacNew) in patients undergoing cardiac rehabilitation.

**Methods:**

The MacNew was filled out by 5692 inpatients. We analysed acceptance (number of missing values), ceiling and floor effects, reliability (Cronbach’s α), factor structure (confirmatory factor analysis), construct validity (correlation with a generic health-related quality of life instrument), and sensitivity to change.

**Results:**

Two items had more than 7% missing data. We observed neither floor nor ceiling effects. Cronbach’s α of the scales ranged from 0.78 (physical scale) to 0.95 (global scale). Confirmatory factor analysis failed to reproduce the proposed factor structure (CFI = 0.882; TLI = 0.871; RMSEA = 0.074). We therefore drafted our own model (CFI = 0.932; TLI = 0.921; RMSEA = 0.064), and observed a correlation pattern largely conforming to the hypotheses with a generic health-related quality of life instrument. The effect sizes we noted between the start and end of rehabilitation fell between 0.66 and 0.74; at the 6-month follow-up they ranged from 0.69 to 0.92.

**Conclusions:**

The German version of the MacNew Heart Disease Health-related Quality of Life Questionnaire is a suitable instrument with which to measure the impairment experienced by individuals with heart disease during inpatient cardiologic rehabilitation. The social and the global scale must be interpreted cautiously.

## Background

Quality of life (QOL) is of central importance in daily life, and most people wish to maximise or at least maintain it. The growing emphasis on the significance of QOL is apparent in public-health evaluation research in which the patient’s QOL is considered the endpoint [1]. In health-care economics, health-related QOL is reflected in quality-adjusted life years (QALYs*)* as utility in cost-utility analyses [2]. It is in evaluating complex therapeutic situations like those frequently encountered in medical rehabilitation that health-related quality of life (HRQOL) from the patient’s perspective has demonstrated particular advantages over “harder” biological endpoints (e.g.[3,4]). Multidisciplinary rehabilitation with its biopsychosocial disease model does not just aim to change certain physical functions, rather it aims to improve or regain the patient’s activity and participatory abilities according to the International Classification of Functioning, Disability and Health (ICF) of the WHO [5]. It is thus not surprising that HRQOL has become a widely-acknowledged and valued parameter in medical rehabilitation evaluations [6].

Improving HRQOL also makes obvious sense in cardiac rehabilitation [7,8]. However, to enable such therapeutic results to be accurately and reliably measured, we need psychometrically-tested means of collecting such data. Important to consider is that there are several types of instruments in HRQOL which differ in being either generic or disease-specific [1]. The disease-specific instruments address complaints that are characteristic to certain diseases, whereas generic instruments enquire about aspects beyond the specific illness. The decision as to which version to employ depends on the nature of the procedure in question regarding that particular query. In other words, generic questionnaires permit more than just comparison among various diseases – they enable effects to be identified on fields not directly related to the given illness. Disease-specific instruments are however considered more sensitive, and thus better suited to measuring changes in disease-related aspects [9-11].

While there are several generic instruments of proven quality in the German rehabilitation system (i.e. IRES-3 [12] and the German SF-36 version [13]), there is a lack of German-language, disease-specific instruments applicable in cardiac rehabilitation. The MacNew Heart Disease Health-related Quality of Life questionnaire (MacNew) [14] is just such an instrument. The English-language MacNew [15,16] evolved from the interview version of the Quality of Life after Myocardial Infarction instrument [17,18]. The reliability of the English version shows Cronbach’s α values between 0.93 and 0.95 and is thus considered very good [19]. Its validity and sensitivity to change are generally satisfactory. There are indications that the original three-factor model does not provide optimal fit, which is why Dempster et al. [20] suggest using a five-factor model. There is reference data describing clinical significance for the English version [21] – the authors designate a mean difference of 0.5 points as a “minimal clinically-important difference”.

The MacNew is now available in a German-language version and was used within various projects (e.g.[22-24]). However, to our knowledge, psychometric reanalyses were done only within the Austrian and Swiss health-care systems in small patient cohorts (68 < N < 200) [14,25-27]. To use this MacNew questionnaire within the German health-care system, we need to investigate its psychometric properties in the German population. Moreover, this psychometric examination should involve a large patient cohort so as to yield results both convincing and representative of rehabilitating patients in the German system. It was the aim of this analysis to determine the psychometric properties of the MacNew questionnaire in a large cohort of patients in rehabilitation by testing acceptance, ceiling and floor effects, reliability (internal consistency), factor structure, construct validity, and sensitivity to change.

## Methods

### Patients

The questionnaires were given only to patients able and willing to fill them out and who had provided informed consent. 8654 patients from 37 cardiac rehabilitation clinics throughout Germany were asked to participate, of whom 5692 agreed. The percentage of patients that did not fill out the questionnaire (decliners) was 34.2%. The most important reason for exclusion was refusal to participate (N = 745), followed by cognitive or physical limitations (N = 575), language problems (N = 263), transfer to another institution (N = 59), and discontinuing rehabilitation (N = 29). 149 patients gave other reasons for not participating, and 1374 patients gave no reason for refusing enrolment (patients could name more than one reason). Thus we had data at hand for our final analysis from 5692 patients in rehabilitation who participated in the quality assurance programme of the statutory health insurance funds in medical rehabilitation between autumn 2004 and spring 2007 (QS-Reha®-Verfahren [6,28]). The study has been approved by the ethics committee of the University of Freiburg (approval number 265/2000). Patients were queried at the start (N = 5692, 100%) and at discharge from the rehabilitation centre (N = 5169; 90.8%), as well as six months after having been discharged (N = 3663; 64.4%).

Table 1 provides information on the study patients*.* N = 523 patients left the rehabilitation centre and thus the study between the start and end of rehabilitation: N = 97 patients refused to continue participating, N = 24 quit rehabilitation, N = 67 patients were transferred, and N = 9 patients stopped for other reasons. A total of N = 339 patients in rehabilitation gave no reason whatsoever for dropping out of the study. Here, too, they could have provided more than one reason.

**Table 1:** Cohort description

	**Rehabilitation-start**	**Rehabilitation-end**	**6-monthfollow-up**
N	5692	5169	3663
Age (Mean [SD])	69.8 [7.5]	69.7 [7.4]	69.8 [7.1]
Gender (%)			
female	35.9	35.6	34.2
male	64.1	64.4	65.8
Retired (%)			
yes	96.5	95.4	96.8
no	3.5	3.4	3.2
Highest educational level (%)			
<10 years	66.7	66.0	63.5
≥10 years	32.3	32.8	35.6
no school qualification	1.2	1.2	0.9
Case groups (%)			
coronary artery disease	78.1	78.9	80.0
angina pectoris	7.8	8.0	8.9
cardiomyopathy	3.7	3.8	3.5
myocarditis	0.4	0.4	0.3
arterial hypertension	50.4	50.6	50.5
heart valve disease	7.8	7.9	7.8
other cardiac disease	20.5	20.5	19.4
Acute events and operations (%)			
heart attack	42.2	42.7	42.6
PTCA	36.7	37.6	38.0
CABG	40.8	40.6	41.7
valve repair	17.8	17.9	17.3
Sypmtom onset (%)			
acute event	24.6	24.8	25.5
<1 year	35.4	35.3	35.6
1–2 years	11.0	10.9	10.9
3–5 years	9.7	9.5	9.3
6–10 years	5.8	5.9	5.3
>10 years	8.4	8.3	8.2
cannot be assessed	5.1	5.2	5.2

Abbreviations: *PTCA* = percutaneous transluminal coronary angioplasty; *CABG* = coronary artery bypass graft.

### Instruments

Höfer et al. published the German version of the MacNew in 2003 [14]. This questionnaire contains 27 items summarised as emotional scale (12 items), social scale (11 items) and physical scale (5 Items) with item 6 being assigned both to the emotional and the physical scale. All the items taken together make up a global scale. The English version [16] contains 26 items, as the item "sexual intercourse" has been omitted from scoring due to high numbers of missing values. However, it has mostly been included in the questionnaire as it could provide important information for therapeutic decisions. The allocation of the items to scales differs in the English version as well, although a factor analysis revealed their loading pattern to be similar to that in the German version [27]. In the English version, the emotional scale has 14 items, the physical 10 items, and the social scale 3 items with item 26 being allocated to the physical scale as well as to the social scale. The MacNew poses questions on complaints becoming apparent during the previous two weeks that are associated with heart disease. Patients complete the self-administered MacNew using a seven point Likert scale (1 = minor to 7 = severe). The range of values of the resulting scales also ranges from one to seven.

We also employed the IRES-3 [29], a questionnaire used to document generic QOL whose 144 items are accommodated in 27 scales forming eight dimensions. The eight dimensions of the IRES are: “physical health“, “pain“, “ability to function in daily life“, “ability to function at work“, “emotional well-being”, “social integration“, “health behaviour“ and “dealing with the disease“.

### Analyses

#### Practicability and distribution characteristics

We investigated the acceptance of the MacNew by referring to the percentage of missing values per item and scale. This was followed by testing ceiling and floor effects per item and scale level. We noted a ceiling or floor effect whenever over 50% of the answers fell into the highest or lowest of the seven categories, respectively.

### *Reliability*

To test internal consistency, we calculated Cronbach´s α [30] in each of the MacNew scales. We then examined the corrected item-scale correlation. An item was defined as selective when it correlated sufficiently with the total score of the scale: coefficients from 0.30 were classified as “moderate“, those from 0.50 as “good“.

### *Factor structure*

To test the unidimensionality of the MacNew scales we carried out a confirmatory factor analysis. This involved first imputing the missing data using the Expectation-Maximation-Algorithm in NORM software [31] and then testing the validity of the three-factor model of the MacNew [27]. We relied on the Comparative Fit Index (CFI), Tucker-Lewis Index (TLI) and root mean square error of approximation (RMSEA) as indications of model quality. CFI and TLI values >0.90 indicate a good fit, RMSEA values <0.10 suggest a moderate fit; values <0.05 are a good fit [32]*.* In addition to testing the original German model of the MacNew [27], we tested each scale on its own. To cross-validate, we halved the sample at random. We developed a new model from the first partial sample after eliminating certain items (1. more than 10% missing values, 2. ceiling or floor effect) by using explorative factor analysis (principal component analysis with VARIMAX rotation). In so doing, an item in a scale was assigned when its assignation was understandable from its content and it loaded on one factor (> = 0.50 on one factor, <0.40 on all the others). The resulting factor model was then cross-validated in the other half of the sample via confirmatory factor analysis. Measurement errors were not allowed to intercorrelate.

### *Construct validity*

Construct validity was tested using IRES-3 dimensions [12] and the Pearson correlation. We hypothesised that the MacNew emotional scale would correlate significantly and closely (r > 0.50, cf. [33]) with the IRES “emotional well-being” dimension, that the correlation with the other IRES dimensions would be at most moderately close (between 0.30 and 0.50, cf. [33]*,* and that the MacNew’s social scale would correlate significantly and closely with the IRES “social integration“ dimension, the physical scale of the MacNew with the IRES dimension “physical health”, and the MacNew’s global scale with the IRES total “Rehab-Status“ score. To assess whether the correlation differences are significantly different we calculated Steiger’s test.

### *Sensitivity to change*

It is imperative when measuring outcomes that the instrument used be capable of demonstrating change during rehabilitation. To be able to make such demands on the MacNew, we calculated the standardized effect sizes [34] between the start and discharge of rehabilitation, and between the start of rehabilitation and six months after discharge. The standardized effect size is the quotient from the difference between the means of two values (i.e., start and end of rehabilitation) and the standard deviation of the value at baseline (rehabilitation start). The strength of these effects can be taken to indicate the sensitivity to change of the questionnaire. As in Cohen [33], effect sizes of 0.20 were considered “small“, around 0.50 “medium”, and >0.80 were deemed “large”.

All our statistical analyses were carried out using PASW Statistics 19 software, while the AMOS 19 was used to do the confirmatory factor analysis.

## Results

### Practicability and distribution characteristics

Our practicability and distribution results are illustrated in Tables 2 and 3. The portion of missing values was 9.5% in one item (item 17 "limitations in sports because of a heart problem") and 26.2% in another (item 17 "limitations in sexual intercourse"). All the other items revealed a portion of missing values between 1.9 and 6.3%. None of the scales in the MacNew showed more than 12.1% missing values, and all the answer categories apply to all items. We observed no signs of floor or ceiling effects in any of the items or scales in the MacNew.

**Table 2:** Item properties of the MacNew questionnaire (N = 5692)

**Item**		**Missing values(%)**	**Mean**	**SD**	**Floor effect(%)**	**Ceiling effect(%)**	**Corrected Item-Total Correlation**	**Scale**
1.	Frustrated	2.2	4.27	1.74	6.5	13.0	0.73	E
2.	Worthless	2.5	5.01	1.81	4.0	29.6	0.75	E
3.	Confident	3.2	4.22	1.74	6.5	11.1	0.60	E
4.	Down in the dumps	1.9	4.71	1.72	3.6	20.2	0.84	E
5.	Relaxed	2.5	3.61	1.79	11.2	5.9	0.65	E
6.	Worn out	2.0	3.55	1.63	10.6	4.8	0.69/0.62	E/P
7.	Happy with personal life	2.5	3.84	1.31	5.6	3.6	0.66	E
8.	Restless	3.0	4.63	1.63	2.8	15.6	0.74	E
9.	Short of breath	2.6	4.59	1.72	3.8	17.7	0.56	P
10.	Tearful	2.1	5.26	1.63	1.6	34.0	0.68	E
11.	More dependent	3.4	4.78	1.79	4.7	23.0	0.61	S
12.	Social activities	3.7	3.93	2.06	17.5	14.5	0.67	S
13.	Others/less confidence in you	4.1	5.88	1.42	1.1	48.0	0.51	E
14.	Chest pain	3.1	4.63	1.80	5.7	20.3	0.52	P
15.	Lack self-confidence	3.4	5.08	1.60	1.7	25.6	0.74	E
16.	Aching legs	2.5	4.20	1.84	7.6	15.6	0.54	P
17.	Sports/exercise limited	9.5	3.44	2.09	25.6	10.5	0.70	S
18.	Frightened	3.0	4.61	1.74	4.3	18.7	0.75	E
19.	Dizzy/lightheaded	2.9	4.94	1.65	2.6	24.7	0.55	P
20.	Restricted or limited	4.3	3.83	1.95	14.4	10.3	0.77	S
21.	Unsure about exercise	6.3	3.92	1.87	12.4	11.3	0.62	S
22.	Overprotective family	4.5	3.43	2.08	23.7	13.1	0.41	S
23.	Burden on others	3.8	5.71	1.59	2.1	46.6	0.49	S
24.	Excluded	3.8	4.82	2.04	9.1	30.6	0.70	S
25.	Unable to socialize	3.5	5.49	1.77	3.4	43.2	0.65	S
26.	Physically restricted	4.1	3.80	1.98	15.3	10.8	0.76	S
27.	Sexual intercourse	26.2	3.91	2.45	29.3	26.7	0.45	S

Range for all items 1–7. SD = standard deviation, E = emotional scale, S = social Scale, P = physical scale.

**Table 3:** Scale properties of the MacNew questionnaire

**Scale**	**Number of Items**	**Missing values(%)**	**Mean**	**SD**	**Floor-effect(%)**	**Ceiling-effect(%)**	**Cronbach’s α**
Emotional	12	5.7	4.56	1.24	0.9	6.3	0.93
Social	11	12.1	4.29	1.35	2.0	7.1	0.89
Physical	5	7.4	4.39	1.26	1.5	4.4	0.78
Global	27	11.1	4.47	1.15	0.5	4.9	0.95

Range for all scales 1–7. SD = standard deviation, Min = Minimum, Max = Maximum.

### Reliability

We obtained internal consistency values of α = 0.93 in the emotional, α = 0.89 in the social, and α = 0.78 in the physical scale (Table 3). The sensitivity coefficients were over 0.50 for all items except items 22, 23 and 27 (Table 2), which belong to the social scale.

### Factor structure

The fit indices of the confirmatory factor analysis indicate a moderate model fit for the original German three-factor model [27] (Table 4).

**Table 4:** Model fit of the various factor models

**Model**	**CFI (>.90)**	**TLI (>.90)**	**RMSEA (<.05)**
original German 3-factor model (Höfer [27])	0.882	0.871	0.074
* Emotional scale* (Höfer)*	0.947	0.936	0.083
* Social scale* (Höfer)*	0.889	0.861	0.113
* Physical scale* (Höfer)*	0.961	0.935	0.090
our own 4-factor model (*cross-validation*)	0.932	0.921	0.064

* This are fit indices for the separate scales of the original German 3-factor model (Höfer).

When regarding the scales of this model individually, one notes that the fit indices of the emotional and physical scales suggest a good model fit, while the model fit of the social scale is poor*.* Developing a new model we removed item 27 “sexual intercourse”, as it had shown 26.2% missing values. No item had to be removed because of ceiling or floor effects. In the next step we omitted items 11 “more dependent”, 15 “lack self-confidence”, 17 “sports/exercise limited”, *20* “restricted or limited”, *24* “excluded” and 25 “unable to socialise”, as they had failed to load convincingly on any one of the factors.

Table 5 illustrates our final model. The fit indices of this model demonstrate good model fit in the cross-validation (Table 4). The scale characteristics of our modified model are found in Table 6.

**Table 5:** Factor loadings of the MacNew items for our modified factor model

**Item**		**Emotional**	**Participation**	**Perception of others**	**Symptoms**
1.	Frustrated	0.745			
2.	Worthless	0.701			
3.	Confident	0.649			
4.	Down in the dumps	0.791			
5.	Relaxed	0.725			
6.	Worn out	0.627			
7.	Happy with personal life	0.663			
8.	Restless	0.710			
9.	Short of breath				0.664
10.	Tearful	0.600			
12.	Social activities		0.627		
13.	Others/less confidence in you			0.745	
14.	Chest pain				0.609
16.	Aching legs				0.592
18.	Frightened	0.596			
19.	Dizzy/lightheaded				0.546
21.	Unsure about exercise		0.615		
22.	Overprotective family		0.556		
23.	Burden on others			0.701	
26.	Physically restricted		0.768		
*Variance explained*		*22.6%*	*16.1%*	*10.9%*	*10.5%*

**Table 6:** Scale properties of the MacNew questionnaire for our modified factor structure

**Scale**	**Number of Items**	**Missing values(%)**	**Mean**	**SD**	**Min**	**Max**	**Floor effect(%)**	**Ceiling effect(%)**	**Cronbach’s α**
Emotional	10	4.7	4.37	1.28	1	7	2.0	5.7	0.92
Participation	4	4.5	3.77	1.49	1	7	7.8	5.0	0.73
Perception of others	2	6.4	5.80	1.31	1	7	0.8	34.4	0.69
Symptoms	4	3.2	4.59	1.29	1	7	1.1	8.1	0.72
Global	20	6.9	4.44	1.12	1	7	0.5	3.8	0.93

### Construct validity

Testing construct validity, we observed that the MacNew emotional scale correlated at r = 0.73 with the IRES-3 “emotional well-being” dimension (Table 7). The correlation value between the MacNew social scale and IRES “social integration“ dimension was r = 0.16, and r = 0.68 between the MacNew physical scale and IRES dimension “physical health“. The MacNew’s global scale and the IRES total “Rehab-Status“ score correlated at r = 0.73. Thus we observed correlation patterns that conform to our hypotheses in all but the social scale. All the relevant correlation differences were significantly different from each other according to Steiger’s test.

**Table 7:** Pearson correlation of the MacNew scales with the IRES-3 dimensions

**IRES dimensions**	**MacNew emotional scale**	**MacNew social scale**	**MacNew physical scale**	**MacNew global scale**
Physical health	0.54^**^	0.54^**^	0.68^**^	0.63^**^
Health behaviour	0.34^**^	0.22^**^	0.26^**^	0.30^**^
Ability to function day to day	0.49^**^	0.53^**^	0.61^**^	0.58^**^
Ability to function at work	0.42^**^	0.34^**^	0.30^**^	0.40^**^
Emotional well-being	0.73^**^	0.54^**^	0.63^**^	0.70^**^
Dealing with the disease	0.55^**^	0.45^**^	0.45^**^	0.55^**^
Sociale integration	0.33^**^	0.16^**^	0.23^**^	0.27^**^
Pain	0.36^**^	0.30^**^	0.45^**^	0.39^**^
Total score „Rehab-Status“	0.70^**^	0.59^**^	0.71^**^	0.73^**^

** p < 0.001.

### Sensitivity to change

The standardized effect size of each of the scales between the start and end of rehabilitation lay between 0.66 (emotional and physical scale) and 0.69 (social scale). The effect size of the global scale between these two timepoints was 0.74 (Table 8). The effect size between the start of rehabilitation and at 6-month follow-up ranged from 0.62 (physical scale) and 0.92 (social scale). The global scale’s effect size here was 0.86 (Table 9).

**Table 8:** Effect sizes from rehabilitation-start to rehabilitation-end

	**N**	**Rehabilitation-start**		**Rehabilitation-end**		Effect size*
		**M**	**SD**	**M**	**SD**	
Emotional scale	4789	4.59	1.23	5.39	1.12	0.66
Social scale	4322	4.30	1.35	5.24	1.17	0.69
Physical scale	4622	4.41	1.26	5.25	1.12	0.66
Global scale	4426	4.48	1.14	5.33	1.04	0.74

*Effect size = (M_rehabilitation-end_ - M_rehabilitation-start_)/SD_rehabilitation start_.

**Table 9:** Effect sizes from rehabilitation-start to 6-month follow-up

	**N**	**Rehabilitation-start**		**6-month follow-up**		Effect size*
		**M**	**SD**	**M**	**SD**	
Emotional scale	3354	4.62	1.22	5.46	1.11	0.69
Social scale	3016	4.30	1.35	5.54	1.13	0.92
Physical scale	3225	4.46	1.25	5.23	1.16	0.62
Global scale	3066	4.50	1.14	5.48	1.04	0.86

*Effect size = (M_6-month follow-up_ - M_rehabilitation-start_)/SD_rehabilitation start_.

## Discussion

Most of the MacNew’s missing values vary in terms of their agreement with previous reports by between 1.9 and 6.3% [16]. We observed nearly 10% missing values in conjunction with item 17 “sports/exercise limited”. This relatively high value may have to do with the fact that most patients are in an acute-care facility at the start of rehabilitation when they first fill out the questionnaire, and thus cannot engage in sports or take exercise. This supposition is supported by the observation that the missing values at discharge from rehabilitation amounted to just 3.8%. Nearly a third of all patients did not answer item 27 “sexual intercourse”. This is probably because this is a taboo subject many patients are reluctant to address. Another reason for the high rate of missing values here could be the patient’s disease course, and the infeasibility of having sex with a partner while in hospital, a pattern reflected in our data. The percentage of this item’s missing values at discharge from rehabilitation was still 31.7%, sinking dramatically to 19.6% at the 6-month follow-up and thus lower than at the start of rehabilitation, even though it remains relatively high compared to the other items. Yet it may also be an artefact, since we can assume that only those patients particularly motivated to take part in the study answered the 6-month follow-up, thereby providing fewer missing values at that point in time. However, this possibility is not confirmed by our data, as the percentage of missing values at that point in time is nearly as high as at the start of rehabilitation. The number of missing values in the “sports/exercise” item falls within an acceptable range, while the “sexual intercourse” value is much too high. Perhaps these items should be removed from the questionnaire to reduce the total number of missing values. Yet one should make such a step dependent on the purpose for which the MacNew is being administered: if it is being employed to evaluate a therapy, one should minimise the production of missing values as far as possible. If however the aim is to identify areas requiring intervention, such items can provide helpful hints, and even if patients fail to answer them, we can still query them about important aspects of daily life. The percentage of missing values on the scale level falls within an acceptable range, and neither floor nor ceiling effects were evident on the scale or item levels. Thus one can consider the MacNew to be an acceptable and comprehensible questionnaire.

Cronbach’s α value fell within an acceptable range in the MacNew factor model proposed by Höfer et al [27]. Moreover, the corrected item-total correlation coefficients showed good selectivity in nearly all items – being in the moderate range in only three items in the social scale*.* This reinforces the reliability of MacNew also. However, to convincingly evaluate reliability, the unidimensionality of the scales must be verified [35], which we did in a confirmatory factor analysis. As the fit indices reveal an only moderate model fit, the unidimensionality is questionable, thus the MacNew’s reliability cannot be definitively assessed. One cause of this suboptimal fit of the three-factor model could be the contextual heterogeneity of the social scale, which first suggests its corrected item-total correlation coefficients, and secondly, that items are being captured here that address physical limitations (i.e. items 17 and 26) together with items whose content is more social in nature (i.e. items 23 und 25). Our investigation of each scale via confirmatory factor analysis has strengthened our suspicion that the cause of moderate model fit can be found in the social scale. While the fit indices show a good model fit in the emotional and the physical scales, those of the social scale reveal an unsatisfactory model fit. These model fit issues have been addressed by several other investigators: Höfer et al. [19] described various item allocations as depending on the languages. If one considers just the English version, there are two different models. Thus Dempster et al. developed a five-factor model from what was originally a poorly-fitting three-factor model [16]. They accommodate 25 items of the MacNew in five scales: emotional, restrictions, physical symptoms, perception of others, and social functioning. Yet even their five-factor model [20] was not entirely verified in our confirmatory factor analysis (results not reported here). In fact, it performed worse in our analysis than the three-factor model [27]. There also seem to be problems with the factor model in the German version of the MacNew. Höfer et al. [25-27] report three different loading patterns in three different analyses, wherein the items do not load unequivocally on the scales. In their analysis in 2005, the item “worn out” loads on the social scale at 0.59 and at 0.61 in the emotional scale. In the original German version, this item is assigned to the emotional scale.

All in all, there seems to be a lack of consensus regarding the MacNew’s factorial quality. We therefore attempted to develop a new factor model for the MacNew using the aforementioned procedures, and arrived at a four-factor solution containing 20 items incorporated in the emotional, participation, perception of others, and symptoms scales. In naming the scales, we follow the example set by Dempster et al. [20] in every instance except for the social scale, which we have named “participation scale” as does the ICF; in our opinion this designation describes the content of those items more accurately. The fit indices of our model offer good model fit, or at least a better fit than is possible in the original German version. Cronbach’s α is somewhat smaller in our model than that in the original German version. Since our new model reveals a good model fit, we can assume at least acceptable accommodation. A limitation of our new model is that the remaining psychometric properties have not yet been assessed. Moreover, whoever uses our model must also question whether an instrument of such brevity is adequate for the job at hand. The fact that the perception of others scale contains only two items is admittedly problematic. Yet the advantage is that our model is more economical. In short, we believe that all of the factor models reported thus far are less than optimal – improvement is especially needed in the social scale. Here, it would be worthwhile to integrate new items reflecting participation more globally as in the ICF, or which reveal limitations in social activities as in the IRES questionnaire. Both would require the production of new items, however. Until this has been exercised, we can only recommend use of the physical and emotional scales. It is not possible to interpret the social scale unequivocally, as long as there is such uncertainty as to what it actually captures. The global scale could be used anyway as the items of the MacNew reflect the broad understanding of health as defined by the WHO.

We can consider the construct validity as verified except for that of the social scale. The correlation patterns of the MacNew scales with those of the IRES dimensions conform to our hypotheses. The poor correlation between the MacNew social scale and “social integration“ IRES dimension may be caused by the fact that the two scales capture different contextual aspects. In the MacNew, the social scale addresses largely corporal aspects, as in item 17 *“*sports/exercise limited” or item 26 *“*physically restricted”, while the IRES dimension exclusively addresses the patient’s social support. A further sign of this is the close correlation between the MacNew scale and the IRES dimension “physical health”. The poor performance of the social scale in terms of construct validity serves to highlight its need for revision. Yet the overall construct validity of the MacNew can be considered proven. It is not possible to compare our results with others in the English- and German-speaking literature, as they used the SF-36 to test construct validity (e.g. [36]). However, even correlations with the SF-36 reveal hypothesis-conforming correlations with the scales in the MacNew (e.g. [20] for the English version). They observed no signs of inadequate construct validity in the social scale.

MacNew is also capable of capturing short and mid-term changes; moderately-sized effects are apparent between the start of rehabilitation and its conclusion, or at the 6-month follow-up. As we used a more conservative effect size measure, this is a hint for the ability of the MacNew to assess changes. Overall, our results regarding the psychometric properties resemble those from other investigators testing the quality of both the English and German versions of the MacNew (see [19]).

A limitation of our study is that our results cannot be extrapolated entirely, as made obvious by our study cohort’s recruitment: 34% of those qualifying for study participation could not be enrolled for various reasons. This corresponds roughly with usual drop-out rates in similar investigations. Thus we cannot claim that our study patients are entirely representative. Moreover, our patients were all insured by the statutory health insurance, and they were all inpatients. Nevertheless, our study cohort does not differ in age or gender substantially from cohorts in other investigations examining psychometric properties of the MacNew (e.g. [14,26]). What is positive is that our patients came from throughout Germany and from different clinics, which in turn supports the claim of their being sufficiently representative. We are unable to make any claims as to the test-retest reliability of the MacNew within the framework of this study design. Höfer et al. [27], however, report good test-retest reliability with a test-retest correlation of over 0.80.

## Conclusions

All in all, we can consider the German version of the MacNew Heart Disease Health-related Quality of Life questionnaire to be a suitable instrument with which to document the impairment experienced by individuals with heart disease during inpatient cardiac rehabilitation. However, the factor structure of the social and global scales remains somewhat problematic. Therefore the social and the global scale must be interpreted cautiously.

## Abbreviations

CFI, Comparative Fit Index; HRQOL, Health Related Quality of Life; ICF, International Classification of Functioning Disability and Health; IRES, “Indikatoren des Reha-Status” a German-language generic HRQOL questionnaire; MacNew, MacNew Heart Disease Health-related Quality of Life Questionnaire; RMSEA, root mean square error of approximation; SF-36, Short Form (36) Health Survey; TLI, Tucker-Lewis Index; QALYs, quality-adjusted life years; QOL, Quality of Life; WHO, World Health Organization.

## Competing interests

The authors declare that they have no competing interests.

## Authors’ contributions

LG performed data analysis, interpreted the data, and drafted the manuscript. EF was involved in study concept and design, acquisition of the data, data interpretation, manuscript drafting and revision of the manuscript. WHJ was involved in study concept and design, and revision of the manuscript. All authors approved the final manuscript.

## References

[B1] Fayers P Machin D Quality of Life The Assessment, Analysis and Interpretation of Patient-reported Outcomes 2007 2 Wiley,

[B2] Marra CA Marion SA Guh DP Najafzadeh M Wolfe F Esdaile JM Clarke AE Gignac MA Anis AH Not all “quality-adjusted life years” are equal J Clin Epidemiol 2007 60 616 624 10.1016/j.jclinepi.2006.09.006 17493521

[B3] Eldar R Methodology Matters - XIII. Quality of care in rehabilitation medicine Int J Qual Health Care 1999 11 73 79 10.1093/intqhc/11.1.73 10411292

[B4] Sato D Kaneda K Wakabayashi H Nomura T Comparison two-year effects of once-weekly and twice-weekly water exercise on health-related quality of life of community-dwelling frail elderly people at a day-service facility Disability & Rehabilitation 2009 31 84 93 10.1080/09638280701817552 18608400

[B5] Stucki G Cieza A Melvin J The International Classification of Functioning, Disability and Health: a unifying model for the conceptual description of the rehabilitation strategy J Rehabilitation Med 2007 39 279 285 10.2340/16501977-0041 17468799

[B6] Farin E Follert P Gerdes N Jäckel WH Thalau J Quality assessment in rehabilitation centres: the indicator system “Quality Profile.” Disability & Rehabilitation 2004 26 1096 1104 10.1080/09638280410001711441 15371035

[B7] Izawa K Hirano Y Yamada S Oka K Omiya K Iijima S Improvement in Physiological Outcomes and Health-Related Quality of Life Following Cardiac Rehabilitation in Patients With Acute Myocardial Infarction Circ J 2004 68 315 320 10.1253/circj.68.315 15056827

[B8] Shephard RJ Franklin B Changes in the quality of life: a major goal of cardiac rehabilitation J Cardiopulmonary Rehabilitation and Prevention 2001 21 189 10.1097/00008483-200107000-00001 11508178

[B9] Cooper JK Kohlmann T Michael JA Haffer SC Stevic M Health outcomes. New quality measure for Medicare Int J Qual Health Care 2001 13 9 16 10.1093/intqhc/13.1.9 11330450

[B10] Guyatt GH Feeny D Patrick DL Measuring health-related quality of life Ann Intern Med 1993 118 622 629 8452328 10.7326/0003-4819-118-8-199304150-00009

[B11] Post MWM Gerritsen J Diederiks JPM De Witte LP Measuring health status of people who are wheelchair-dependent: validity of the Sickness Impact Profile 68 and the Nottingham Health Profile Disability & Rehabilitation 2001 23 245 253 10.1080/096382801750110874 11336097

[B12] Bührlen B Gerdes N Jäckel WH Entwicklung und psychometrische Testung eines Patientenfragebogens für die medizinische Rehabilitation (IRES-3) Rehabilitation 2005 44 63 74 10.1055/s-2004-834687 15789288

[B13] Bullinger M Kirchberger I Der SF-36 Fragebogen zum Gesundheitszustand 1998 1 Hogrefe, Göttingen Bern Toronto Seattle

[B14] Höfer S Benzer W Schüssler G von Steinbüchel N Oldridge NB Health-related quality of life in patients with coronary artery disease treated for angina: validity and reliability of German translations of two specific questionnaires Quality of Life Res 2003 12 199 212 10.1023/A:1022272620947 12639066

[B15] Lim LL-Y Valenti LA Knapp JC Dobson AJ Plotnikoff R Higginbotham N Heller RF A self-administered quality-of-life questionnaire after acute myocardial infarction J Clin Epidemiol 1993 46 1249 1256 10.1016/0895-4356(93)90089-J 8229102

[B16] Valenti LA Lim LL-Y Heller RF Knapp J An improved questionnaire for assessing quality of life after acute myocardial infarction Quality of Life Res 1996 5 151 161 10.1007/BF00435980 8901378

[B17] Hillers TK Guyatt GH Oldridge NB Crowe J Willan A Griffith L Feeny D Quality of life after myocardial infarction J Clin Epidemiol 1994 47 1287 1296 10.1016/0895-4356(94)90134-1 7722565

[B18] Oldridge NB Guyatt GH Jones N Crowe J Singer J Feeny D McKelvie R Runions J Streiner D Torrance G Effects on quality of life with comprehensive rehabilitation after acute myocardial infarction Am J Cardiol 1991 67 1084 1089 10.1016/0002-9149(91)90870-Q 2024598

[B19] Höfer S Lim LL-Y Guyatt GH Oldridge NB The MacNew Heart Disease health-related quality of life instrument: a summary Health and Quality of Life Outcomes 2004 2 3 10.1186/1477-7525-2-3 14713315 PMC341459

[B20] Dempster M Donnelly M O’Loughlin C The validity of the MacNew Quality of Life in heart disease questionnaire Health and Quality of Life Outcomes 2004 2 6 10.1186/1477-7525-2-6 14738566 PMC333433

[B21] Dixon T Lim LL-Y Oldridge NB The MacNew heart disease health-related quality of life instrument: Reference data for users Quality of Life Res 2002 11 173 183 10.1023/A:1015005109731 12018740

[B22] Meder M Farin E Gesundheitsbewertungen bei Patienten mit chronisch-ischämischer Herzkrankheit Die Rehabilitation 2011 50 222 231 10.1055/s-0030-1263120 21626474

[B23] Oldridge N Saner H McGee HM The Euro Cardio-QoL Project An international study to develop a core heart disease health-related quality of life questionnaire, the HeartQoL Eur J Cardiovasc Prevention & Rehabilitation 2005 12 87 94 10.1097/01.hjr.0000159408.05180.0e 15785293

[B24] Schweikert B Hahmann H Leidl R Validation of the EuroQol questionnaire in cardiac rehabilitation Heart 2006 92 62 67 10.1136/hrt.2004.052787 15797936 PMC1860985

[B25] Höfer S Schmid JP Frick M Benzer W Laimer H Oldridge NB Saner H Psychometric properties of the MacNew heart disease health-related quality of life instrument in patients with heart failure J Eval Clin Pract 2008 14 500 10.1111/j.1365-2753.2007.00905.x 18462292

[B26] Höfer S Anelli-Monti M Berger T Hintringer F Oldridge NB Benzer W Psychometric Properties of an Established Heart Disease Specific Health-related Quality of Life Questionnaire for Pacemaker Patients Qual Life Res 2005 14 1937 1942 10.1007/s11136-005-4347-9 16155781

[B27] Höfer S Benzer W Brandt D Laimer H Schmid P Bernardo A Oldridge NB MacNew Heart Disease Lebensqualitätsfragebogennach Herzinfarkt: Die deutsche Version Z Klin Psychol Psychother 2004 33 270 280 10.1026/1616-3443.33.4.270

[B28] Farin E Jäckel WH Schalaster V Das Qualitätssicherungsverfahren der GKV in der medizinischen Rehabilitation: Ergebnisse und Weiterentwicklung Gesundheitswesen 2009 71 163 174 10.1055/s-0028-1119382 19288432

[B29] Frey C Bührlen B Gerdes N Jäckel WH Handbuch zum IRES-3: Indikatoren des Reha-Status 2007 Version 3 mit IRES 24, Roderer, S

[B30] Cronbach LJ Coefficient alpha and the internal structure of tests Psychometrika 1951 16 297 334 10.1007/BF02310555

[B31] Schafer JL Analysis of Incomplete Multivariate Data 1997 Crc Pr Inc, London

[B32] Hu L-Bentler PM Cutoff Criteria for Fit Indexes in Covariance Structure Analysis: Conventional Criteria Versus New Alternatives Struct Equ Model 1999 6 1 10.1080/10705519909540118

[B33] Cohen J Statistical Power Analysis for the Behavioral Sciences 1988 2 Revised, Lawrence Erlbaum Assoc Inc

[B34] Kazis LE Anderson JJ Meenan RF Effect sizes for interpreting changes in health status Med Care 1989 27 S178 S189 10.1097/00005650-198903001-00015 2646488

[B35] Schmitt N Uses and abuses of coefficient alpha Psychol Assess 1996 8 350 353

[B36] Oldridge NB Perkins A Hodes Z Comparison of three heart disease specific health-related quality of life instruments Monaldi Arch Chest Dis 2002 58 10 18 12693064

